# Sudden deterioration due to intra-tumoral hemorrhage of ependymoma of the fourth ventricle in a child during a flight: a case report

**DOI:** 10.1186/1752-1947-4-143

**Published:** 2010-05-20

**Authors:** Ali Mahdavi, Nima Baradaran, Farideh Nejat, Mostafa El Khashab, Maryam Monajemzadeh

**Affiliations:** 1 Department of Neurosurgery, Children's Hospital Medical Center, Tehran University of Medical Sciences, Tehran, Iran; 2 Department of Neurosurgery, Hackensack University Medical Center, New Jersey, USA; 3 Department of Pathology, Children's Hospital Medical Center, Tehran University of Medical Sciences, Tehran, Iran

## Abstract

**Introduction:**

To the best of our knowledge, the association between air travel and intra-tumoral hemorrhage in pediatric populations has never been described previously.

**Case presentation:**

We report the case of a two-and-a-half-year-old Caucasian, Iranian boy with a hemorrhaging brain tumor. He had a posterior fossa midline mass and severe hydrocephalus. He had been shunted for hydrocephalus four weeks earlier and was subsequently referred to our center for further treatment. The hemorrhage occurred in an infra-tentorial ependymoma, precipitated by an approximately 700-mile air journey at a maximum altitude of 25,000 feet.

**Conclusions:**

A pre-existing intra-cranial mass lesion diminishes the ability of the brain to accommodate the mild environmental disturbances caused by hypercarbia, increased venous pressure and reduced cerebral blood flow during long air journeys. This is supported by a literature review, based on our current knowledge of physiological changes during air travel.

## Introduction

Hemorrhage into brain neoplasms is a relatively uncommon but not a rare occurrence with obvious relevance to the neurosurgeon. In general, about 5% to 10% of all brain tumors develop hemorrhage of some type. The tissue type of the tumor itself is clearly related to its propensity to bleed, as metastatic lesions are known to carry a high risk of hemorrhage. Of the primary brain tumors, glioblastoma appears to be the most common source of intra-cerebral hemorrhage. Oligodendrogliomas, astrocytomas, ependymomas and medulloblastomas have also been associated with intra-cranial hemorrhage. Less commonly, benign tumors such as pituitary adenomas and meningiomas have also been demonstrated to bleed [1].

Several pathophysiological factors have been described to account for spontaneous hemorrhage within brain tumors, including coagulation defects and vascular abnormalities [2]. However, only a few cases of hemorrhage precipitated by air travel have been reported. Some physiological changes are well-documented to occur during commercial flights. These include decreased barometric pressure, cerebral hypoperfusion or hypoxemia, mild degrees of hypercarbia and local hemostatic abnormalities which may be associated with hemorrhage within cerebral tumors [3,4].

To the best of our knowledge, two adult cases of hemorrhage within brain tumors after air travel have been described before [4]. The association between flight and intra-tumoral hemorrhage in the pediatric population has not been described previously. Potential biological mechanisms underlying this association are also discussed.

## Case presentation

**Figure 1:**
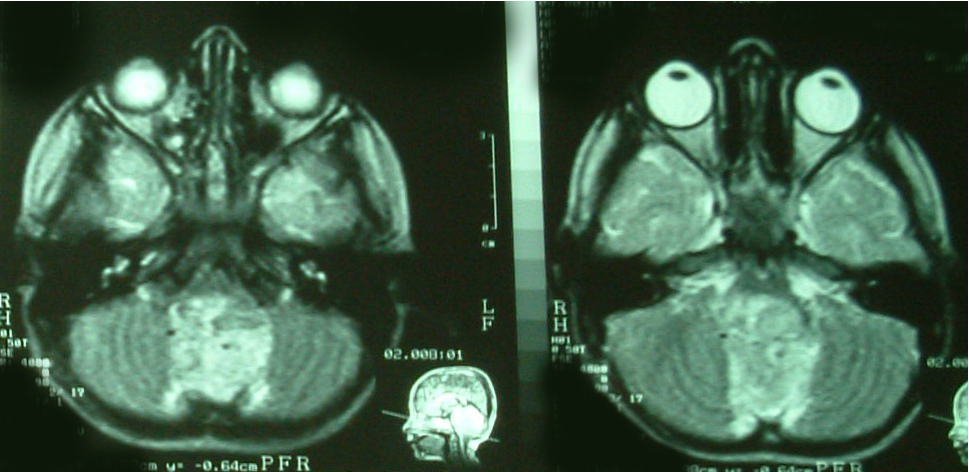
**Brain magnetic resonance imaging carried out before the last admission reveals hyperintense mass in T2-weighted image filling the fourth ventricle without any evidence of hemorrhage**.

A two-and-a-half-year-old Caucasian boy with posterior fossa midline mass and severe hydrocephalus was brought to our emergency room immediately after completing a 700-mile air flight. He was generally well before the flight suffering from moderate ataxic gait and lower cranial nerve involvement. About 40 minutes into the flight, he had developed severe headache and retractable vomiting leading to rapid onset loss of consciousness. He had been shunted for hydrocephalus four weeks earlier and was subsequently referred to our center for further treatment. Brain computed tomography (CT) and magnetic resonance imaging (MRI) were performed before referral, which had demonstrated satisfactory decompression of the hydrocephalus without hemorrhage signal inside the tumor bed (Figure 1).

On admission, he was unconscious, unable to follow commands, but able to localize painful stimuli. He had apneustic breathing but bilateral reactive pupils were retained. Over the next few hours, he developed severe hypoxia that necessitated mechanical ventilation. A brain ultrasound confirmed normal-sized ventricles but without any new information about the posterior fossa mass. Given the fact that cerebrospinal fluid shunting had been carried out several weeks earlier, upward herniation was assumed an unlikely event. He was admitted to the pediatric intensive care unit (PICU) and revealed that he was given high-dose dexamethasone and supportive therapy with the initial diagnosis of brain stem compression due to the tumor mass. Three days after admission, his condition had not changed and he underwent resection of the tumor through a midline sub-occipital approach.

**Figure 2:**
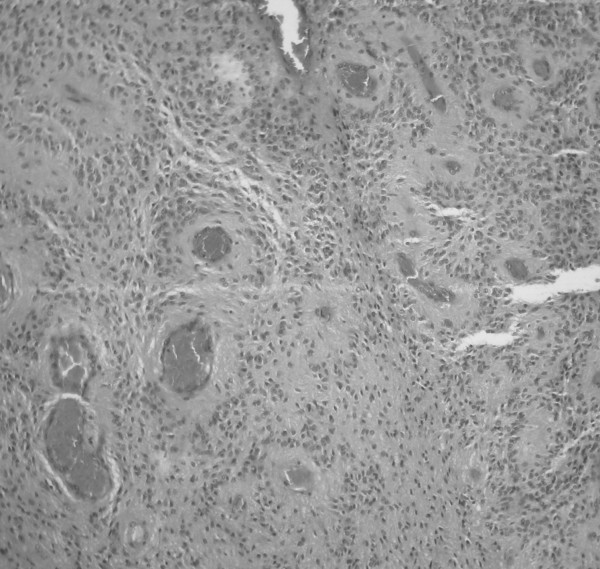
**The pathological specimen shows cellular and vascular tumor with diagnostic features of the ependymoma such as the presence of prominent nucleus-free zones around blood vessels (perivascular pseudorosettes) (hematoxylin and eosin, original magnification ×40)**.

**Figure 3:**
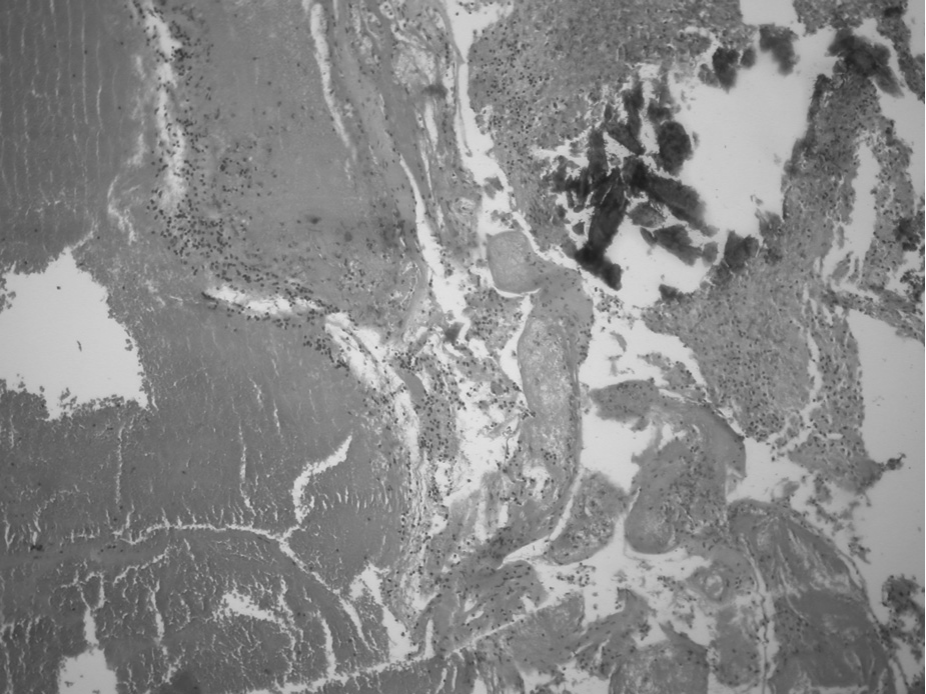
**Large area of hemorrhage (left side), necrosis (right and bottom) and calcification (right and upper side), original magnification ×100**.

During the operation, the posterior fossa was very tense through the exposed field. There was blood-tinged cerebellum in the midline with a small subdural clot. The tumor was soft, reddish-gray, amenable to suction and highly vascular containing a large area of hemorrhage (Figures 2 and 3). It was almost completely resected except for a thin layer attached to the inferior triangle of the fourth ventricle floor. The boy had an uneventful early recovery post-operatively. He was breathing independently one day after surgery and gradually regained consciousness and was able to obey commands three days later. His long-term swallowing difficulty persisted post-operatively so feeding was begun through a nasogastric tube. Histopathological examination of the tumor revealed an anaplastic ependymoma. Our patient was seen by our pediatric oncologist for adjuvant chemotherapy. Six months later, he underwent standard cranial radiotherapy. One year after surgery, he is tumor-free with mild ataxia and with no lower cranial nerve problems.

## Discussion

Children with posterior fossa ependymomas most often have slowly evolving signs and/or symptoms of intra-cranial hypertension or cerebellar dysfunction. However, there are rare occasions where the tumor can lead to a severe illness. Hemorrhage into these tumors is not rare and might present as apoplexy [5-7]. The main pathophysiologies of hemorrhage into tumors include structural abnormalities in tumor vessels, tumor invasion into cerebral vessel walls, tumor or brain necrosis, and coagulation defects, either related to systemic cancer or iatrogenically induced [2].

However, the pathophysiological mechanisms by which air travel could predispose the tumors to bleed are not fully-understood. Decreased barometric pressure, hypoxemia, and local hemostatic abnormalities are well-known physiological changes during air travel.

Commercial airplanes cruising at typical altitudes of 30,000 to 40,000 feet partially pressurize their cabins to the atmospheric pressures found at 5000 to 8000 feet, or 552 to 632 mmHg. Due to the resultant decrease in the partial pressure of oxygen in the inspired air, blood oxygen saturation levels of as low as 85% may be reached [8]. These changes in oxygen levels can cause considerable effects on hemostasis in humans. Infants and young children are particularly susceptible to hypoxemic episodes [9]. Due to this relative hypoxemia, patients who have recently suffered strokes by cerebrovascular accidents are often advised not to fly in order to prevent additional ischemic neuronal loss. Goldberg and Hirschfeld suggest that the relative hypoxemia experienced during the flight may result in differential ischemic changes in tumor tissue, and with an already tenuous blood supply delivered through the thin-walled, non-autoregulating vasculature, tumor necrosis and respective hemorrhage into the necrotic tissue are more likely [4].

Additionally, local tissue ischemia may be a manifestation of decreased perfusion due to hypovolemia. The most commonly reported in-flight malady is syncope. Venous pooling of blood in the lower extremities as a result of prolonged sitting combined with dehydration from low cabin humidity and poor fluid intake have been reported to contribute to intra-vascular volume depletion. However, cerebral vasculature may be able to compensate for this through normal autoregulatory mechanisms, which are lacking in the tumor vascular structure [4].

An alternative hypothesis concerns the role of decreased cabin pressure resulting in an increase in tumor venous pressure due to transmission of mildly increased intra-abdominal pressure through the inferior vena cava and cranial dural sinuses. At a cabin pressure of 575 mmHg, gas expands to 132% of its baseline volume at sea level [10]. Expansion of intestinal gas may have brought about a mild elevation of intra-abdominal pressure with consequent venous rupture or thrombosis within the intra-cranial tumor. The increased transmural pressure across tumor blood vessels due to the rapid lowering of intra-cranial pressure has been implicated in tumors that bleed after ventricular shunts or drainage [4]. The decreased atmospheric pressure in the airplane cabin may have caused a similar effect that induces a transmural pressure difference within blood vessels and the surrounding environment, resulting in tumor hemorrhage.

Another mechanism may be related to increased levels of inspired CO_2 _in commercial flights. Cabin air undergoes a degree of recycling as well as exchange with atmospheric air. This process leads to an increasing inspired fraction of CO_2 _levels in aircraft cabins during flight. US federal aviation law specifies a CO_2 _level of less than 0.5% in the cabin air [10]. However, this mild degree of hypercapnia may lead to the well-documented phenomenon of cerebral vasodilation [8,10] and consequent tumor vessel rupture.

The sudden onset of neurological deficits in our patient, who had previously been well and with a functioning shunt, suggest bleeding in the pre-existing intra-cranial tumor, which was confirmed intra-operatively. The possible mechanisms of bleeding in the highly vascular and potentially hemorrhage-prone tumor in our patient during flight can be more than a coincidence and might be related to fluctuation of cabin CO_2_, oxygen levels and interior pressure. Therefore, it seems reasonable to assume that these physiological changes pose an additional risk of hemorrhage into brain tumors in decompensated individuals.

## Conclusions

Although obviously rare, this case together with previously described cases suggest that it is reasonable to caution patients with a known intra-cranial mass lesion about the possible risks of commercial flight. Prophylactic medications such as steroid agents, acetazolamide, and supplementary O_2 _may be considered for these patients during air travel. In spite of all the suggested mechanisms, we believe that in order to offer thorough guidelines for patient care during flight, further studies need to be carried out with respect to the effects of air travel on health.

## Competing interests

The authors declare that they have no competing interests.

## Authors' contributions

AM, NB and FN managed our patient and collected and interpreted our patient data regarding the disease and the possible mechanism of hemorrhage during flight. ME helped during management and was a major contributor in writing the manuscript. MM performed the histological examination of the tumor sample. All authors read and approved the final manuscript.

## Consent

Written informed consent was obtained from the patient's parents for publication of this case report and any accompanying images. A copy of the written consent is available for review by the Editor-in-Chief of this journal.
